# Racial and ethnic disparities in the refusal of surgical treatment in women 40 years and older with breast cancer in the USA between 2010 and 2017

**DOI:** 10.1007/s10549-022-06653-w

**Published:** 2022-06-24

**Authors:** Pierre Fwelo, Zenab I. Yusuf, Abigail Adjei, Gabriel Huynh, Xianglin L. Du

**Affiliations:** 1 Department of Epidemiology, Human Genetics & Environmental Sciences, UTHealth School of Public Health, 7000 Fannin St., Suite 2052-4, Houston, TX 77030 USA; 2 Department of Management, Policy and Community Health, UTHealth School of Public Health, Houston, TX USA; 3 Department of Psychiatry, Baylor College of Medicine, Houston, TX USA

**Keywords:** Surgical treatment, Racial disparities, Breast cancer, Survival, Subtypes

## Abstract

**Purpose:**

Although surgical resection is the main modality of treatment for breast cancer, some patients elect to refuse the recommended surgery. We assessed racial and ethnic differences in women 40 years and older who received or refused to receive surgical treatment for breast cancer in the USA and whether racial disparities in mortality were affected by their differences in the prevalence of refusal for surgical treatment.

**Methods:**

We studied 277,127 women with breast cancer using the Surveillance, Epidemiology, and End Results (SEER) data and performed multivariable logistic regressions to investigate the association between surgery status of breast cancer and race/ethnicity. Additionally, we performed Cox regression analyses to determine the predictors of mortality outcomes.

**Results:**

Of 277,127 patients with breast cancer, 1468 (0.53%) refused to receive the recommended surgical treatment in our cohort. Non-Hispanic Black women were 112% more likely to refuse the recommended surgical treatment for breast cancer compared to their non-Hispanic White counterparts [adjusted odds ratio: 2.12, 95% confidence interval (CI) 1.82–2.47]. Women who underwent breast-conserving surgery [hazards ratio (HR) 0.15, 95% CI 0.13–0.16] and mastectomy (HR 0.21, 95% CI 0.18–0.23) had lower hazard ratios of mortality as compared to women who refused the recommended treatment after adjusting for covariates.

**Conclusion:**

Race/ethnicity was associated with refusal for the recommended surgery, especially among non-Hispanic Black women. Also, surgery refusal was associated with a higher risk of all-cause and breast cancer-related mortality. These disparities stress the need to tailor interventions aimed at raising awareness of the importance of following physician recommendations among minorities.

**Supplementary Information:**

The online version contains supplementary material available at 10.1007/s10549-022-06653-w.

## Introduction

Breast cancer is the most prevalent cancer and the second leading cause of cancer-related deaths after lung cancer among U.S. women [1–4]. In 2021, it is estimated that approximately 281,550 new cases of invasive breast cancer will be diagnosed and 43,600 women will die from breast cancer in the USA. Approximately, 3.8 million U.S. women currently live with a history of breast cancer [2, 3]. Additionally, nearly 1 in 8 (13%) women in the USA will be diagnosed with invasive breast cancer and 1 in 39 (2.6%) will die of it [1, 2, 4, 5]. Breast cancer incidence rates are the highest among non-Hispanic (NH) Whites (130.8 per 100,000 women) and NH Blacks (126.7 per 100,000 women). NH Black women have the highest recorded breast cancer death rate (28.4 deaths per 100,000) and are more likely to die from breast cancer at any age as compared to other ethnic groups [2, 6].

It is well documented that the main modality of treatment for breast cancer is surgical resection either in the form of lumpectomy (breast-conserving surgery—BCS) or modified mastectomy (removal of the entire breast) based on pathological characteristics, which is often followed by adjuvant treatment, such as radiation therapy and/or chemotherapy [2, 7, 8]. However, corroboration from controlled clinical trials showed that there has been an increasing concern that cancer patients in the USA do not receive the full benefit of effective and appropriate cancer treatment [9, 10].

Although surgical treatment has been proven effective, research showed that some patients develop various setbacks. Post-surgery patients may develop declining psychosocial functioning with respect to their quality of life, cancer-related distress, sexual dysfunction, body image dysmorphism, anxiety, and depression, hence affecting surgery refusal rates [7, 11–13]. Previous studies have also found age, race, marital status, health insurance, and income level to be associated with surgery refusal, with NH Black patients being less likely to receive breast cancer surgical treatment [7, 14–19]. However, those previous studies focused on data prior to 2014. Information and data on patients diagnosed in more recent years are needed. Also, the extent to which these disparities result from differences in cancer molecular subtype characteristics among a large and ethnically diverse cohort of older women remains unexplored.

The acknowledgment of treatment refusal has led to various quality improvement measures to better direct strategies to improve surgical utilization, patient satisfaction, and cancer care outcome [20–22]. Understanding which patients are potentially likely not to abide by the recommended treatment and when the treatment is most likely to be curative would be of great clinical benefit. This information should also help address, identify, and reduce cancer care outcome disparities.

Therefore, this study aimed to determine if there were racial and ethnic disparities in the prevalence of refusals for definitive surgeries (i.e., mastectomy or lumpectomy) in women with early-stage breast cancer who were eligible and recommended for surgeries, whether the refusal of receiving the recommended surgeries was associated with an increased risk of mortality, and whether racial disparities in mortality were affected by their differences in the prevalence of refusal for surgical treatment.

## Methods

### Data source, study design, and patient selection

This study utilized the Surveillance, Epidemiology, and End Results (SEER) database. SEER is a cancer registry program supported by the National Cancer Institute (NCI) that aims to provide reliable incidence and survival data [23]. We selected the SEER research plus data for 18 registries in November 2020 database, which covers nearly 28% of the U.S. population [24]. A detailed description of the database and data collection can be found elsewhere [23]. The study subjects were de-identified from an existing public-use dataset and there was no patient contact; thus, the study is exempt from an Institutional Review Board’s review.

We conducted a retrospective cohort study using secondary data of women with a primary diagnosis of breast cancer at age 40 years and older between 2010 and 2017 in the SEER areas of the USA. We focused on women 40 years and older to capture women at risk of being diagnosed with breast cancer based on the American Cancer Society breast cancer screening recommendation [25]. We investigated the racial and ethnic disparities between women who received surgery to treat their breast cancer and those who refused the surgical recommendation. Additionally, we assessed the differences in breast cancer survival outcomes by surgery type (recommended but refused surgery versus BCS or Mastectomy).

**Fig. 1:**
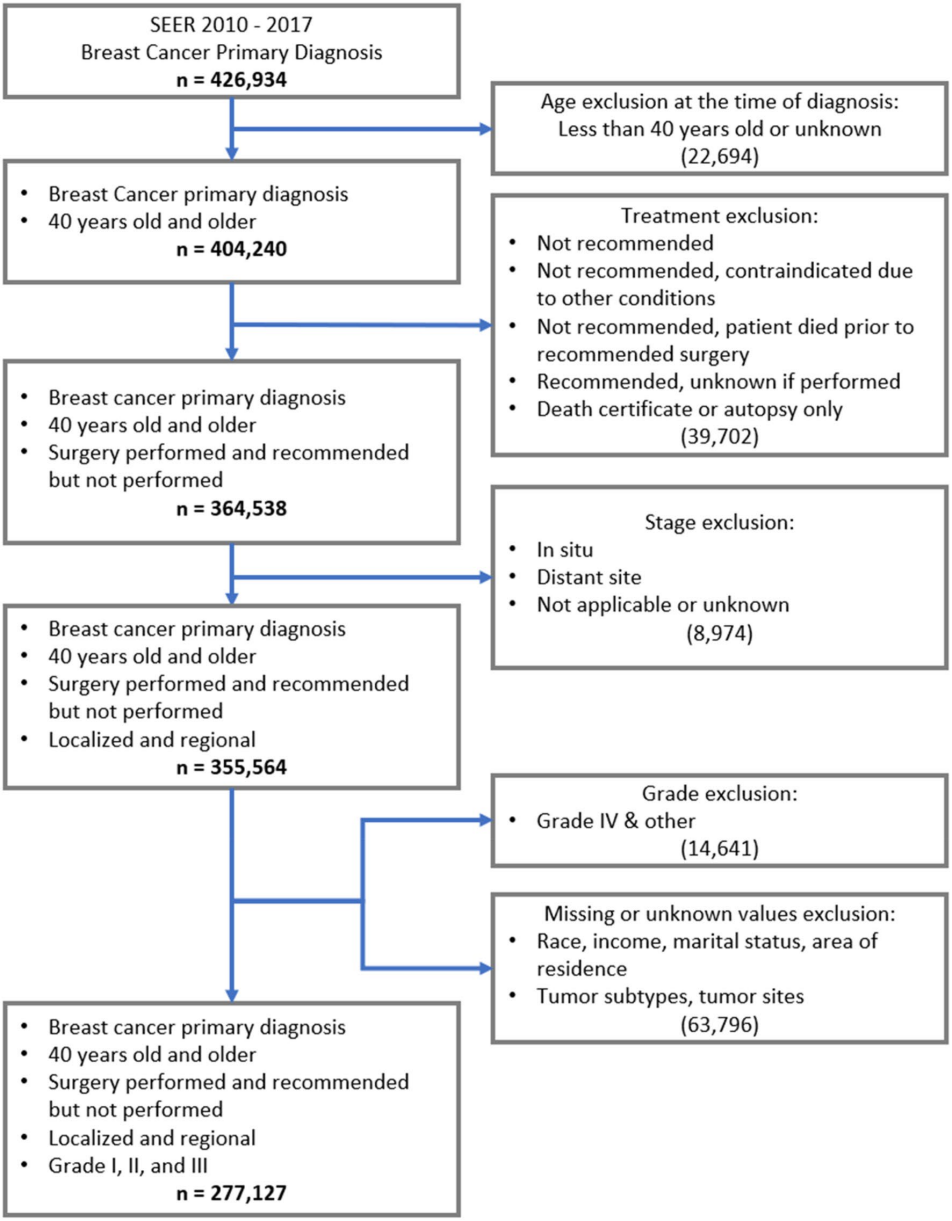
Sample selection

We used the SEER Stat software 8.3.8 to identify all women 40 years or older with breast cancer as the first primary diagnosis from 2010 to 2017 (*n* = 404,240). We included eligible patients for whom surgery was recommended and performed or for whom surgery was not performed due to patients’ refusal. We excluded patients with grade IV tumor, distant tumor stage, or unknown status on race, income, marital status, area of residence, tumor sites, breast cancer subtypes, and ineligible for surgery (Fig. 1). The new sample size was 277,127 and then 267,999 following logistic and cox regression analyses when observations with missing values were dropped.

### Study variables

#### Main exposure

Race/ethnicity was classified into non-Hispanic White, non-Hispanic Black, Hispanic, and other. For the secondary analysis, surgery type was categorized into breast-conserving surgery (BCS) and mastectomy, and no surgery (i.e., patient refused) was assessed as a predictor of death. Those ineligible cases for surgery were excluded (Fig. 1).

#### Outcomes

*Surgery treatment* was the main outcome of interest and dichotomized as “surgery performed” and “surgery recommended but not performed, patient refused.” We also looked at death from any causes, death from breast cancer, and 5-year survival as secondary outcomes. *All-cause mortality* was a dichotomous variable (yes/no). Patients who died of any causes at the end of the study were categorized as “yes,” and those who did not were categorized as “no.” *Breast cancer-related death* was a dichotomous variable. Patients who died of breast cancer at the end of the study were categorized as “yes,” and those who died of other causes were “censored.”

#### Survival months

For overall mortality, survival time was calculated from the date of diagnosis to the date of death or the date of last follow-up (December 31, 2017) as indicated in the SEER registry. For the breast cancer-related mortality, survival time was calculated from the date of diagnosis to the date of breast cancer-related death or the date of last follow-up as indicated in the SEER registry.

#### Tumor characteristics and adjuvant treatment

The following tumor characteristics and treatment variables were obtained from SEER and assessed based on findings from previous studies [7, 8, 11, 14]: year of diagnosis (2010, 2011, 2012, 2013, 2014, 2015, 2016, and 2017); tumor subtype (luminal A, luminal B, HER2 enriched, and triple negative); tumor grade (Grade I, grade II, and grade III); tumor site (nipple, central portion of the breast, upper-inner quadrant of the breast, lower-inner quadrant of the breast, upper-outer quadrant of the breast, lower-outer quadrant of the breast, and axillary tail of the breast); tumor stage (localized only, regional with direct extension, regional with lymph nodes only, and regional with both direct extension and lymph nodes); and adjuvant treatment: radiotherapy (yes/no) and chemotherapy (yes/no). We also assessed pathological inflammatory breast cancer (IBC). Women with pathological IBC were defined as breast cancer cases coded with ICD-O-3 histologic code 8530 (yes/no).

#### Sociodemographic characteristics

Age at diagnosis was categorized into 40–49, 50–59, 60–69, 70–79, and 80+. Marital status was classified as married, unmarried/domestic partner, divorced, widowed, separated, and never married. Median household income inflation adjusted to 2019 was grouped into < $35 k; $35,000–44,999, $ 45,000–54,999, $ 55,000–64,999, $65,000–74,999, and ≥ $75 k at the census tract level. Area of residence was organized into counties in metropolitan areas greater than 1 million, counties in metropolitan areas of 250 k to 1 million, counties in metropolitan areas less than 250 k, nonmetropolitan counties adjacent to a metropolitan area, and nonmetropolitan counties non-adjacent to a metropolitan area.

### Statistical analysis

We performed descriptive statistics to review the characteristics of the cohort stratified by race/ethnicity and by surgery status. We used Pearson’s Chi-square test to determine if the differences were statistically significant. We performed crude and multivariable logistic regression to assess the association between the surgical treatment of breast cancer and race/ethnicity.

We performed crude and three adjusted Cox proportional hazards regressions to determine the association between surgery types and race/ethnicity and the mortality outcomes (death and survival). The first model assessed the association between surgery type and mortality outcomes by adjusting for sociodemographic factors; the second model adjusted for sociodemographic and tumor factors; and the third model adjusted for radiation therapy and chemotherapy in addition to those variables in the second model.

We also undertook six sensitivity analyses to assess the impact of different age cut-off and pathological inflammatory breast cancer on the results. In the first sensitivity analysis, we recalculated the adjusted odds ratios (aORs) for refusal of the recommended surgery, excluding patients 40–49 years old (17.35% of main cohort). The second sensitivity analysis excluded patients 40–59 (43.62% of main cohort) and recalculated aORs for refusal of the recommended surgery. In the third sensitivity analysis, we excluded patients with pathological inflammatory breast cancer (0.08% of main cohort) and recalculated the aORs for refusal of the recommended surgery. In the fourth sensitivity analysis, we recalculated the adjusted hazard ratios (aHRs) for breast cancer-related mortality, excluding patients 40–49 years old (17.35% of main cohort). The fifth sensitivity analysis excluded patients 40–59 (43.62% of main cohort) and recalculated aHRs for breast cancer-related mortality. In the sixth sensitivity analysis, we excluded patients with pathological inflammatory breast cancer (0.08% of main cohort) and recalculated the aHRs breast cancer-related mortality. The results of these sensitivity analyses are shown in Supplemental Tables S1–S6. SAS version 9.4 and STATA 16.1 were used to perform the analyses.

## Results

### Sociodemographic characteristics

A description of the sample baseline characteristics stratified by race/ethnicity is provided in Table 1. The proportion of patients with triple-negative breast cancer was 20.38% among non-Hispanic Black patients, while the proportion was 9.15% and 10.88% among non-Hispanic and Hispanic patients, respectively. The proportion of patients with Grade 3 tumors was 43.78% among non-Hispanic Black patients, 26.78% among non-Hispanic White, and 33.56% among Hispanic patients. The descriptive analysis also showed differences in marital status within racial groups. Non-Hispanic Black had the lowest percentage of being married or with a domestic partner (37.80%), while it was 61.99% and 58.39% among non-Hispanic Whites and Hispanics, respectively.

**Table 1:** Sociodemographic and cancer characteristics of women 40 and above in the USA by Race/Ethnicity (2010–2017)

Variable	Number of patients (*n* = 277,127)	Non-Hispanic White (*n* = 191,777)	Non-Hispanic Black (*n* = 28,250)	Hispanic (All races) (*n* = 30,813)	Other (*n* = 26,287)	Pearson Chi-square
Surgery						86.4692***
Patients with surgery recommended but not performed, patients refused	1,486	938 (0.49)	257 (0.91)	140 (0.45)	151 (0.57)	
Patients who received surgery	275,641	190,839 (99.51)	27,993 (99.09)	30,673 (99.55)	26,136 (99.43)	
Subtypes						4.6exp(3)***
Luminal A	209,996	149,932 (78.18)	17,973 (63.62)	22,437 (72.82)	19,654 (74.77)	
Luminal B	27,478	17,776 (9.27)	3,064 (10.85)	3,585 (11.63)	3,053 (11.61)	
HER2 enriched	10,838	6,530 (3.40)	1,456 (5.15)	1,438 (4.67)	1,414 (5.38)	
Triple negative	28,815	17,539 (9.15)	5,757 (20.38)	3,353 (10.88)	2,166 (8.24)	
Age at diagnosis						5.7exp(3)***
40–49	48,079	28,312 (14.76)	5,566 (19.70)	7,963 (25.84)	6,238 (23.73)	
50–59	72,810	47,643 (24.84)	8,443 (29.89)	9,225 (29.94)	7,499 (28.53)	
60–69	81,609	58,454 (30.48)	8,031 (28.43)	7,819 (25.38)	7,305 (27.79)	
70–79	51,583	39,045 (20.36)	4,509 (15.96)	4,225 (13.71)	3,804 (14.47)	
80+	23,046	18,323 (9.55)	1,701 (6.02)	1,581 (5.13)	1,441 (5.48)	
Tumor grade						4.2exp(3)***
Grade I; well differentiated	69,674	52,293 (27.27)	4,722 (16.72)	6,675 (21.66)	5,984 (22.76)	
Grade II; moderately differentiated	125,240	88,117 (45.95)	11,160 (39.50)	13,796 (44.77)	12,167 (46.29)	
Grade III; poorly differentiated	82,213	51,367 (26.78)	12,368 (43.78)	10,342 (33.56)	8,136 (30.95)	
Tumor site						367.0890***
Nipple	1,008	647 (0.34)	101 (0.36)	142 (0.46)	118 (0.45)	
Central portion of the breast	14,156	9,912 (5.17)	1,234 (4.37)	1,516 (4.92)	1,494 (5.68)	
Upper-inner quadrant of the breast	40,050	27,181 (14.17)	4,102 (14.52)	4,486 (14.56)	4,281 (16.29)	
Lower-inner quadrant of the breast	17,578	12,006 (6.26)	2,098 (7.43)	1,875 (6.09)	1,599 (6.08)	
Upper-outer quadrant of the breast	108,230	75,762 (39.51)	11,157 (39.49)	11,808 (38.32)	9,503 (36.15)	
Lower-outer quadrant of the breast	23,767	16,572 (8.64)	2,442 (8.64)	2,580 (8.37)	2,173 (8.27)	
Axillary tail of the breast	1,305	885 (0.46)	197 (0.70)	135 (0.44)	88 (0.33)	
Overlapping lesion of the breast	71,033	48,812 (25.45)	6,919 (24.49)	8,271 (26.84)	7,031 (26.75)	
Tumor stage						1.3exp(3)***
Localized only	197,500	139,928 (72.96)	18,501 (65.49)	20,277 (65.81)	18,794 (71.50)	
Regional, direct extension only	3,423	2,437 (1.27)	368 (1.30)	332 (1.08)	286 (1.09)	
Regional, lymph nodes only	68,290	44,318 (23.11)	8,323 (29.46)	9,169 (29.76)	6,480 (24.65)	
Regional, both direct extension and lymph nodes	7,914	5,094 (2.66)	1,058 (3.75)	1,035 (3.36)	727 (2.77)	
Marital status						1.1exp(4)***
Married/Domestic Partner	165,394	118,800 (61.99)	10,678 (37.80)	17,991 (58.39)	17,845 (67.89)	
Divorced	32,038	22,181 (11.57)	4,457 (15.78)	3,456 (11.22)	1,944 (7.40)	
Widowed	37,364	27,269 (14.22)	4,096 (14.50)	3,142 (10.20)	2,857 (10.87)	
Separated	3,073	1,407 (0.73)	681 (2.41)	724 (2.35)	261 (0.99)	
Never married	39,258	22,040 (11.49)	8,338 (29.52)	5,500 (17.85)	3,380 (12.86)	
Year of diagnosis						348.4360***
2010	30,717	22,092 (11.52)	2,999 (10.62)	2,953 (9.58)	2,673 (10.17)	
2011	31,810	22,487 (11.73)	3,110 (11.01)	3,375 (10.95)	2,838 (10.80)	
2012	33,035	23,131 (12.06)	3,415 (12.09)	3,521 (11.43)	2,968 (11.29)	
2013	34,302	23,840 (12.43)	3,480 (12.32)	3,753 (12.18)	3,229 (12.28)	
2014	34,957	24,208 (12.62)	3,629 (12.85)	3,838 (12.46)	3,282 (12.49)	
2015	36,769	25,244 (13.16)	3,716 (13.15)	4,210 (13.66)	3,599 (13.69)	
2016	37,470	25,276 (13.18)	3,899 (13.80)	4,559 (14.80)	3,736 (14.21)	
2017	38,067	25,499 (13.30)	4,002 (14.17)	4,604 (14.94)	3,962 (15.07)	
Median income						1.8exp(4)***
< $35,000	3,797	2,946 (1.54)	645 (2.28)	135 (0.44)	71 (0.27)	
$35,000–$44,999	19,617	13,749 (7.17)	4,795 (16.97)	840 (2.73)	233 (0.89)	
$45,000–$54,999	40,359	29,846 (15.56)	5,508 (19.50)	3,940 (12.79)	1,065 (4.05)	
$55,000–$64,999	66,982	43,241 (22.55)	7,620 (26.97)	10,239 (33.23)	5,882 (22.38)	
$65,000–$74,999	58,796	42,341 (22.08)	4,776 (16.91)	7,209 (23.40)	4,470 (17.00)	
> $75,000	87,576	59,654 (31.11)	4,906 (17.37)	8,450 (27.42)	14,566 (55.41)	
Urban–Rural						6.7exp(3)***
Counties in metropolitan areas greater than 1 million	172,590	112,780 (58.81)	19,349 (68.49)	21,765 (70.64)	18,696 (71.12)	
Counties in metropolitan areas of 250 k to 1 million	58,086	40,567 (21.15)	4,942 (17.49)	6,737 (21.86)	5,840 (22.22)	
Counties in metropolitan areas less than 250 k	19,049	14,817 (7.73)	1,950 (6.90)	1,440 (4.67)	842 (3.20)	
Non-metropolitan counties adjacent to a metropolitan area	15,984	13,759 (7.17)	1,505 (5.33)	499 (1.62)	221 (0.84)	
Non-metropolitan counties non-adjacent to a metropolitan area	11,418	9,854 (5.14)	504 (1.78)	372 (1.21)	688 (2.62)	
Radiation therapy						983.2506***
No	106,188	71,589 (37.33)	10,494 (37.15)	12,957 (42.05)	11,148 (42.41)	
Yes	161,811	114,632 (59.77)	16,559 (58.62)	16,347 (53.05)	14,273 (54.30)	
Missing	9,128	5,556 (2.90)	1,197 (4.24)	1,509 (4.90)	866 (3.29)	
Chemotherapy						3.0exp(3)***
No	171,081	124,213 (64.77)	14,039 (49.70)	17,061 (55.37)	15,768 (59.98)	
Yes	106,046	67,564 (35.23)	14,211 (50.30)	13,752 (44.63)	10,519 (40.02)	
Pathological inflammatory breast cancer						0.074
No	276,913	191,635 (99.93)	28,223 (99.90)	30,781 (99.90)	26,274 (99.95)	
Yes	214	142 (0.07)	27 (0.10)	32 (0.10)	13 (0.05)	

Values are *n* (% of column total). For the *p*-value, *ns* indicates not significant, *p < 0.05, **p < 0.01, ***p < 0.001

*HER2* Human epidermal growth factor receptor 2

A description of the sample baseline characteristics stratified by surgery treatment status is provided in Table 2. Overall, 0.53% of women with breast cancer refused the recommended breast cancer treatment. The surgery refusal rate was higher in non-Hispanic Black women (0.91%) than in non-Hispanic Whites (0.49%), Hispanics (0.45%), and others (0.57%). Chi-square test comparing women who underwent surgery as recommended versus those who did not receive surgery revealed statistically significant differences (*p* < 0.0001) by patient and tumor characteristics. Although non-Hispanic Black patients represented 10.19% of all women with breast cancer, they accounted for 17.29% of breast cancer women who refused to receive the recommended treatment as compared to 10.19% of those who did in our cohort. On the other hand, non-Hispanic White patients accounted for 63.12% of the patients who refused to undergo surgery as prescribed. The proportion of women who refused the recommended surgery were higher for ≥ 80 years and for those who had a localized, luminal A, and grade II moderately differentiated tumor located in the upper-outer quadrant of the breast.

**Table 2:** Sociodemographic and cancer characteristics of women 40 and above in the USA by Surgery status (2010–2017)

Variable	Number of patients (*n* = 277,127)	Patients who received surgery (*n* = 275,641)	Patients with surgery recommended but not performed (*n* = 1486)	Pearson Chi-square
Race and ethnicity				86.4692***
Non-Hispanic White	191,777	190,839 (99.51)	938 (0.49)	
Non-Hispanic Black	28,250	27,993 (99.09)	257 (0.91)	
Hispanic (All races)	30,813	30,673 (99.55)	140 (0.45)	
Other	26,287	26,136 (99.43)	151 (0.57)	
Subtypes				10.2803*
Luminal A	209,996	208,897 (99.48)	1,099 (0.52)	
Luminal B	27,478	27,296 (99.34)	182 (0.66)	
HER2 enriched	10,838	10,775 (99.42)	63 (0.58)	
Triple negative	28,815	28,673 (99.51)	142 (0.49)	
Age at diagnosis				2.3exp(3)***
40–49	48,079	47,940 (99.71)	139 (0.29)	
50–59	72,810	72,571 (99.67)	239 (0.33)	
60–69	81,609	81,362 (99.70)	247 (0.30)	
70–79	51,583	51,355 (99.56)	228 (0.44)	
80+	23,046	22,413 (97.25)	633 (2.75)	
Tumor grade				26.4617***
Grade I; well differentiated	69,674	69,362 (99.55)	312 (0.45)	
Grade II; moderately differentiated	125,240	124,473 (99.39)	767 (0.61)	
Grade III; poorly differentiated	82,213	81,806 (99.50)	407 (0.50)	
Tumor site				49.6519***
Nipple	1,008	1,000 (99.21)	8 (0.79)	
Central portion of the breast	14,156	14,030 (99.11)	126 (0.89)	
Upper-inner quadrant of the breast	40,050	39,842 (99.48)	208 (0.52)	
Lower-inner quadrant of the breast	17,578	17,496 (99.53)	82 (0.47)	
Upper-outer quadrant of the breast	108,230	107,710 (99.52)	520 (0.48)	
Lower-outer quadrant of the breast	23,767	23,656 (99.53)	111 (0.47)	
Axillary tail of the breast	1,305	1,298 (99.46)	7 (0.54)	
Overlapping lesion of the breast	71,033	70,609 (99.40)	424 (0.60)	
Tumor stage				615.9641***
Localized only	197,500	196,538 (99.51)	962 (0.49)	
Regional, direct extension only	3,423	3,311 (96.73)	112 (3.27)	
Regional, lymph nodes only	68,290	67,990 (99.56)	300 (0.44)	
Regional, both direct extension and lymph nodes	7,914	7,802 (98.58)	112 (1.42)	
Marital status				759.8123***
Married	164,397	163,925 (99.71)	472 (0.29)	
Unmarried/Domestic partner	997	996 (99.90)	1 (0.10)	
Divorced	32,038	31,855 (99.43)	183 (0.57)	
Widowed	37,364	36,837 (98.59)	527 (1.41)	
Separated	3,073	3,044 (99.06)	29 (0.94)	
Never married	39,258	38,984 (99.30)	274 (0.70)	
Year of diagnosis				72.0314***
2010	30,717	30,611 (99.65)	106 (0.35)	
2011	31,810	31,678 (99.59)	132 (0.41)	
2012	33,035	32,871 (99.50)	164 (0.50)	
2013	34,302	34,142 (99.53)	160 (0.47)	
2014	34,957	34,783 (99.50)	174 (0.50)	
2015	36,769	36,532 (99.36)	237 (0.64)	
2016	37,470	37,219 (99.33)	251 (0.67)	
2017	38,067	37,805 (99.31)	262 (0.69)	
Median income				15.7957**
< $35,000	3,797	3,780 (99.55)	17 (0.45)	
$35,000–$44,999	19,617	19,525 (99.53)	92 (0.47)	
$45,000–$54,999	40,359	40,130 (99.43)	229 (0.57)	
$55,000–$64,999	66,982	66,674 (99.54)	308 (0.46)	
$65,000–$74,999	58,796	58,476 (99.46)	320 (0.54)	
> $75,000	87,576	87,056 (99.41)	520 (0.59)	
Urban–Rural				6.8024^ ns^
Counties in metropolitan areas greater than 1 million	172,590	171,648 (99.45)	942 (0.55)	
Counties in metropolitan areas of 250 k to 1 million	58,086	57,756 (99.43)	330 (0.57)	
Counties in metropolitan areas less than 250 k	19,049	18,963 (99.55)	86 (0.45)	
Non-metropolitan counties adjacent to a metropolitan area	15,984	15,913 (99.56)	71 (0.44)	
Non-metropolitan counties non-adjacent to a metropolitan area	11,418	11,361 (99.50)	57 (0.50)	
Radiation therapy				2.2exp(3)***
No	106,188	104,747 (98.64)	1,441 (1.36)	
Yes	161,811	161,787 (99.99)	24 (0.01)	
Missing	9,128	9,107 (99.77)	21 (0.23)	
Chemotherapy				579.0282***
No	171,081	169,714 (99.20)	1,367 (0.80)	
Yes	106,046	105,927 (99.89)	119 (0.11)	

Values are n (% of column total). For the *p*-value, *ns* indicates not significant, *p < 0.05, **p < 0.01, ***p < 0.001

*HER2* Human epidermal growth factor receptor 2

### Trend of surgery refusal over time

**Fig. 2:**
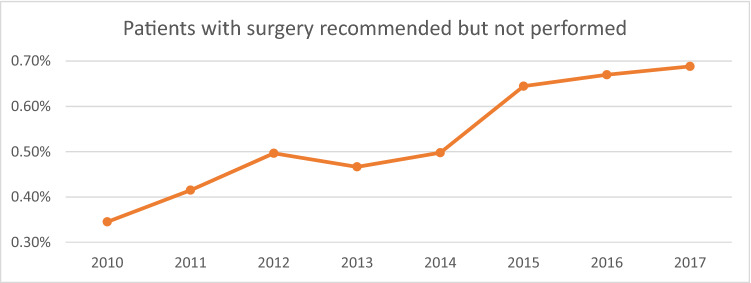
Surgery refusal prevalence over time

Results from Fig. 2 showed an increasing trend in refusing the recommended surgery by year of diagnosis in women with breast cancer. Compared to 2010 (0.35%), the proportion of those who refused surgery increased by about 97% in 2017 (0.69%).

### Primary outcome on surgery refusal

The surgery refusal rate was higher in non-Hispanic Black women (0.91%) than in non-Hispanic Whites (0.49%), Hispanics (0.45%), and others (0.57%). The multivariable logistic regression analysis showed that non-Hispanic Black women had 112% higher odds (AOR: 2.12, 95% CI 1.82–2.47) of refusing the recommended surgery performed compared to non-Hispanic Whites after adjusting for sociodemographic and tumor characteristics as shown in Table 3. Additionally, women aged ≥ 80 years compared to those aged 40–49 years old and those with luminal B, HER2, and triple negative compared to those with luminal A were less likely to undergo the recommended surgery as recommended (Table 3). There was an increasing trend in the proportion of women receiving the recommenced surgery by year of diagnosis from 2013 to 2017. Women residing in metropolitan counties of 250 thousand to 1 million residents had the highest odds (AOR = 1.17, 95% CI 1.02–1.34) of receiving the recommended surgery compared to women residing in counties in metropolitan areas with populations greater than 1 million.

**Table 3:** Crude and multivariable logistic regression analyses of factors associated with refusing to undergo surgery as recommend among US women 40 or above between 2010 and 2017

Variable	Crude Odds Ratio of refusing the recommended surgery (95% Confidence Interval)	Adjusted Odds Ratio of refusing the recommended surgery (95% Confidence Interval)
Race and ethnicity		
Non-Hispanic White	1.00 (reference)	1.00 (reference)
Non-Hispanic Black	1.87 (1.63–2.15)***	2.12 (1.82–2.47)***
Hispanic (All races)	0.93 (0.78–1.11)^ns^	0.97 (0.81–1.17)^ns^
Other	1.18 (0.99–1.397)^ns^	1.18 (0.99–1.42)^ns^
Subtypes		
Luminal A	1.00 (reference)	1.00 (reference)
Luminal B	1.27 (1.08–1.48)**	1.93 (1.62–2.28)***
HER2 enriched	1.11 (0.86–1.43)^ns^	1.64 (1.25–2.16)***
Triple negative	0.94 0(.79–1.12)^ns^	1.36 (1.12–1.66)**
Age at diagnosis		
40–49	1.00 (reference)	1.00 (reference)
50–59	1.14 (0.92–1.4)^ns^	1.15 (0.93–1.43)^ns^
60–69	1.05 (0.85–1.29)^ns^	1.04 (0.84–1.29)^ns^
70–79	1.53 (1.24–1.89)***	0.97 (.77–1.21)^ns^
80+	9.74 (8.10–11.71)***	3.06 (2.48–3.78)***
Tumor grade		
Grade I; well differentiated	1.00 (reference)	1.00 (reference)
Grade II; moderately differentiated	1.37 (1.20–1.56)***	1.31 (1.14–1.49)***
Grade III; poorly differentiated	1.11 (0.954–1.282)^ns^	1.11 (0.94–1.32)^ns^
Tumor site		
Nipple	1.00 (reference)	1.00 (reference)
Central portion of the breast	1.12 (0.55–2.30)^ns^	1.73 (0.83–3.60)^ns^
Upper-inner quadrant of the breast	0.65 (0.32–1.33)^ns^	1.85 (0.89–3.84)^ns^
Lower-inner quadrant of the breast	0.59 (0.28–1.21)^ns^	1.54 (0.73–3.25)^ns^
Upper-outer quadrant of the breast	0.60 (0.30–1.22)^ns^	1.78 (0.87–3.66)^ns^
Lower-outer quadrant of the breast	0.59 (0.9–1.21)^ns^	1.57 (0.75–3.28)^ns^
Axillary tail of the breast	0.67 (0.24–1.86)^ns^	1.75 (0.62–4.99)^ns^
Overlapping lesion of the breast	0.75 (0.37–1.52)^ns^	1.92 (0.93–3.96)^ns^
Tumor stage		
Localized only	1.00 (reference)	1.00 (reference)
Regional, direct extension only	6.91 (5.67–8.43)***	4.2 (3.39–5.21)***
Regional, lymph nodes only	0.90 (0.79–1.03)^ns^	1.57 (1.37–1.81)***
Regional, both direct extension and lymph nodes	2.93 (2.41–3.57)***	4.02 (3.23–4.98)***
Marital status		
Married	1.00 (reference)	1.00 (reference)
Unmarried/Domestic partner	0.35 (0.05–2.48)^ns^	0.4 (0.05–2.84)^ns^
Divorced	2.00 (1.68–2.37)***	1.69 (1.42–2.02)***
Widowed	4.97 (4.39–5.63)***	1.66 (1.43–1.93)***
Separated	3.31 (2.27–4.82)***	2.79 (1.88–4.15)***
Never married	2.44 (2.10–2.83)***	1.88 (1.61–2.19)***
Year of diagnosis		
2010	1.00 (reference)	1.00 (reference)
2011	1.20 (0.93–1.56)^ns^	1.26 (0.97–1.64)^ns^
2012	1.44 (1.13–1.84)**	1.51 (1.18–1.93)**
2013	1.35 (1.058–1.731)*	1.45 (1.13–1.87)**
2014	1.45 (1.134–1.84))**	1.61 (1.25–2.06)***
2015	1.87 (1.489–2.357)***	2.09 (1.66–2.65)***
2016	1.95 (1.55–2.44)***	2.26 (1.79–2.85)***
2017	2.00 (1.60–2.51)***	2.25 (1.79–2.84)***
Median income		
< $35,000	1.00 (reference)	1.00 (reference)
$35,000–$44,999	1.05 (0.62–1.76)^ns^	1.08 (0.632–1.85)^ns^
$45,000–$54,999	1.27 (0.774–2.079)^ns^	1.52 (0.89–2.57))^ns^
$55,000–$64,999	1.03 (0.63–1.676)^ns^	1.20 (0.71–2.06)^ns^
$65,000–$74,999	1.22 (0.746–1.984)^ns^	1.48 (0.86–2.54)^ns^
> $75,000	1.33 (0.82–2.16)^ns^	1.85 (1.08–3.16)*
Urban–Rural		
Counties in metropolitan areas greater than 1 million	1.00 (reference)	1.00 (reference)
Counties in metropolitan areas of 250 k to 1 million	1.04 (0.918–1.181)^ns^	1.17 (1.02–1.337)*
Counties in metropolitan areas less than 250 k	0.83 (0.662–1.031)^ns^	0.91 (.712–1.159)^ns^
Non-metropolitan counties adjacent to a metropolitan area	0.81 0(.638–1.035)^ns^	0.96 (.728–1.265)^ns^
Non-metropolitan counties non-adjacent to a metropolitan area	0.91 (0.70–1.20)^ns^	1.02 (0.75–1.40)^ns^
Radiation therapy		
No	1.00 (reference)	1.00 (reference)
Yes	0.01 (0.01–0.02)***	0.02 (0.01–0.02)***
Chemotherapy		
No	1.00 (reference)	1.00 (reference)
Yes	0.14 (0.12–0.17)***	0.17 (0.13–0.20)***

Values are n (% of column total). For the p-value, ns indicates not significant, *p < 0.05, **p < 0.01, ***p < 0.001

*HER2* Human epidermal growth factor receptor 2

### Secondary outcome on mortality

#### All-cause mortality

Table 4 presents the Cox proportional hazards regression analysis of the association between surgery type and overall mortality adjusted for covariates. To account for the violation of the proportional hazard assumption, the final model treated race, subtypes, tumor grade, and age as time-varying variables by adjusting for the interaction between these variables and the natural log of survival time. Women who underwent BCS (HR 0.34, 95% CI 0.31–0.37) or mastectomy (HR 0.37, 95% CI 0.34–0.40) had significantly lower hazards of death from any cause than women who refused the recommended surgery after adjusting for covariates (model 3). Using non-Hispanic Whites as the reference group, non-Hispanic Black women had the highest crude hazard of all-cause mortality (HR 1.40, 95% CI 1.36–1.45). After adjusting for surgery type and other covariates, the hazard ratio of all-cause mortality decreased but was still significantly higher among NH Black women (HR 1.18, 95% CI 1.01–1.37) in model 3.

**Table 4:** Crude and multivariable Cox regression analyses of factors associated with all-cause mortality and breast cancer-related mortality among US women 40 or above between 2010 and 2017

Variable	Overall Mortality					Breast cancer mortality			
	Crude Proportional Hazard Ratio (95% CI)	Model 1 adjusted proportional Hazard Ratio (95% CI)	Model 2 adjusted proportional Hazard Ratio (95% CI)	Model 3 adjusted proportional Hazard Ratio (95% CI)		Crude Proportional Hazard Ratio (95% CI)	Model 1 adjusted proportional Hazard Ratio (95% CI)	Model2 adjusted proportional Hazard Ratio (95% CI)	Model 3 adjusted proportional Hazard Ratio (95% CI)
Surgery type									
No surgery	1.00 (reference)	1.00 (reference)	1.00 (reference)	1.00 (reference)		1.00 (reference)	1.00 (reference)	1.00 (reference)	1.00 (reference)
Breast conservatory surgery (BCS)	0.12 (0.11–0.13)***	0.23 (0.21–0.25)***	0.26 (0.23–0.28)***	0.34 (0.31–0.37)***		0.07 (0.06–0.08)***	0.10 (0.09–0.12)***	0.13 (0.11–0.14)***	0.14 (0.13–0.16)***
Mastectomy	0.19 (0.18–0.21)***	0.40 (0.37–0.44)***	0.34 (0.31–0.37)***	0.37 (0.34–0.40)***		0.18 (0.16–0.20)***	0.27 (0.25–0.30)***	0.20 (0.18–0.22)***	0.21 (0.19–0.23)***
Race and ethnicity									
Non-Hispanic White	1.00 (reference)	1.00 (reference)	1.00 (reference)	1.00 (reference)		1.00 (reference)	1.00 (reference)	1.00 (reference)	1.00 (reference)
Non-Hispanic Black	1.40 (1.36 -–1.45)***	1.72 (1.49–1.99)*	1.18 (1.02–1.37)*	1.18 (1.01–1.37)*		1.88 (1.80–1.98)***	2.05 (1.64–2.54)***	1.13 (0.90–1.41)^ns^	1.13 (0.90–1.42)^ns^
Hispanic (All races)	0.91 (0.87–0.95)***	0.87 (0.73–1.05)^ns^	0.80 (0.66–0.97)*	0.80 (0.66–0.96)*		1.22 (1.16–1.30)***	0.91 (0.69–1.20)^ns^	0.79 (0.60–1.05)^ns^	0.82 (0.62 –- 1.08)^ns^
Other	0.63 (0.60–0.66)***	0.59 (0.47–0.75)***	0.59 (0.47–0.74)***	0.59 (0.47–0.75)***		0.78 (0.73–0.84)***	0.45 (0.31–0.65)	0.45 (0.31–0.65)	0.47 (0.32–0.68)***
Subtypes									
Luminal A	1.00 (reference)	–	1.00 (reference)	1.00 (reference)		1.00 (reference)	–	1.00 (reference)	1.00 (reference)
Luminal B	1.00 (0.95–1.04)^ns^	–	1.29 (1.06–1.56)**	1.25 (1.03–1.52)*		1.40 (1.31–1.49)***	–	1.66 (1.23–2.27)**	1.56 (1.14–2.15)**
HER2 enriched	1.37 (1.29–1.45)***	–	2.85 (2.27–3.58)***	2.71 (2.15–3.42)***		2.42 (2.25–2.61)***	–	5.69 (4.12–7.86)***	5.31 (3.82–7.40)***
Triple negative	2.21 (2.15–2.28)***	–	5.78 (5.11–6.56)***	5.81 (5.12–6.60)***		4.20 (4.03–4.37)***	–	13.23 (10.92–16.04)***	12.93 (10.62–15.73)***
Age at diagnosis									
40–49	1.00 (reference)	1.00 (reference)	1.00 (reference)	1.00 (reference)		1.00 (reference)	1.00 (reference)	1.00 (reference)	1.00 (reference)
50–59	1.16 (1.11–1.22)***	1.00 (0.81–1.24)^ns^	1.03 (0.83–1.27)^ns^	1.01 (0.81–1.26)^ns^		0.99 (0.94–1.05)^ns^	0.90 (0.68–1.20)^ns^	0.92 (0.69–1.22)ns	0.92 (0.69–1.22)ns
60–69	1.44 (1.38–1.51)***	1.33 (1.08–1.62)***	1.56 (1.27–1.91)***	1.57 (1.27–1.93)***		0.83 (0.79–0.88)***	1.27 (0.95–1.68)^ns^	1.63 (1.23–2.17)**	1.65 (1.23–2.20)**
70–79	2.70 (2.58–2.83)***	2.28 (1.86–2.78)***	2.83 (2.31–3.45)***	2.73 (2.22–3.35)***		1.06 (1.00–1.13)*	3.09 (2.31–4.12)***	4.34 (3.26–5.79)***	4.44 (3.30–5.95)***
80+	7.96 (7.62–8.32)***	5.92 (4.86–7.20)***	6.46 (5.31–7.86)***	5.45 (4.45–6.67)***		2.38 (2.24 –2.54)***	8.44 (6.33–11.27)***	9.60 (7.18–12.82)***	9.97 (7.41–13.41)***
Tumor grade									
Grade I; well differentiated	1.00 (reference)	–	1.00 (reference)	1.00 (reference)		1.00 (reference)	–	1.00 (reference)	1.00 (reference)
Grade II; moderately differentiated	1.32 (1.28–1.37)***	–	1.17 (1.13–1.22)***	1.20 (1.16–1.24)***		2.82 (2.61–3.05)***	–	2.09 (1.93–2.26)***	2.09 (1.93–2.27)***
Grade III; poorly differentiated	2.29 (2.21–2.37)***	–	1.82 (1.75–1.89)***	1.86 (1.79–1.94)***		8.71 (8.09–9.39)***	–	4.51 (4.16–4.89)***	4.42 (4.07–4.80)***
Tumor site									
Nipple	1.00 (reference)	–	1.00 (reference)	1.00 (reference)		1.00 (reference)	–	1.00 (reference)	1.00 (reference)
Central portion of the breast	0.86 (0.73–1.00)^ns^	–	1.05 (0.90–1.23)^ns^	1.11 (0.94–1.30)^ns^		0.97 (0.75–1.25)^ns^	–	1.16 (0.90–1.50)^ns^	1.19 (0.92–1.55)^ns^
Upper-inner quadrant of the breast	0.56 (0.48–0.66)***	–	1.08 (0.92–1.22)^ns^	1.14 (0.97–1.34)^ns^		0.60 (0.46–0.77)***	–	1.25 (0.97–1.61)^ns^	1.28 (0.99–1.65)^ns^
Lower-inner quadrant of the breast	0.64 (0.55–0.75)***	–	1.11 (0.94–1.30)^ns^	1.17 (1.00–1.38)^ns^		0.70 (0.54–0.90)**	–	1.33 (1.03–1.71)*	1.37 (1.05–1.77)*
Upper-outer quadrant of the breast	0.60 (0.51–0.70)***	–	1.05 (0.90–1.22)^ns^	1.11 (0.95–1.30)^ns^		0.66 (0.51–0.84)**	–	1.13 (0.88–1.44)^ns^	1.16 (0.90–1.49)^ns^
Lower-outer quadrant of the breast	0.62 (0.53–0.73)***	–	1.07 (0.91–1.26)^ns^	1.13 (0.96–1.33)^ns^		0.71 (0.55–0.91)**	–	1.22 (0.95–1.57)^ns^	1.24 (0.95–1.60)^ns^
Axillary tail of the breast	0.70 (0.56–0.87)**	–	1.11 (0.89–1.39)^ns^	1.19 (0.95–1.48)^ns^		0.79 (0.56–1.11)^ns^	–	1.11 (0.79–1.57)^ns^	1.16 (0.82–1.64)^ns^
Overlapping lesion of the breast	0.64 (0.55–0.75)***	–	1.09 (0.93–1.27)^ns^	1.15 (0.98–1.35)^ns^		0.69 (0.54–0.88)**	–	1.17 (0.91–1.50)^ns^	1.21 (0.94–1.56)^ns^
Tumor stage									
Localized only	1.00 (reference)	–	1.00 (reference)	1.00 (reference)		1.00 (reference)	–	1.00 (reference)	1.00 (reference)
Regional, direct extension only	4.00 (3.75–4.28)***	–	2.31 (2.17–2.47)***	4.76 (3.67–6.17)***		5.92 (5.34–6.56)***	–	3.35 (3.01–3.73)***	3.35 (3.01–3.73)***
Regional, lymph nodes only	1.75 (1.70–1.79)***	–	1.80 (1.75–1.85)***	2.41 (2.14–2.71)***		3.86 (3.71–4.02)***	–	3.17 (3.04–3.31)***	3.20 (3.06–3.34)***
Regional, both direct extension and lymph nodes	4.80 (4.60–5.00)***	–	3.48 (3.32–3.64)***	6.90 (5.80–8.21)***		11.88 (11.23–- 12.56)***	–	6.88 (6.48–7.31)***	7.11 (6.67–7.58)***
Marital status									
Married/Domestic Partner	1.00 (reference)	1.00 (reference)	1.00 (reference)	1.00 (reference)		1.00 (reference)	1.00 (reference)	1.00 (reference)	1.00 (reference)
Divorced	1.50 (1.45–1.56)***	1.38 (1.33–1.43)***	1.35 (1.30–1.41)***	1.34 (1.29–1.39)***		1.39 (1.32–1.48)***	1.29 (1.22–1.36)***	1.23 (1.16–1.30)***	1.24 (1.17–1.31)***
Widowed	3.35 (3.26–3.45)***	1.51 (1.47–1.56)***	1.47 (1.43–1.52)***	1.45 (1.41–1.50)***		1.97 (1.88–2.07)***	1.35 (1.28–1.43)***	1.28 (1.21–1.35)***	1.29 (1.22–1.36)***
Separated	1.48 (1.32–1.66)***	1.57 (1.40–1.77)***	1.50 (1.35–1.69)***	1.53 (1.36–1.72)***		1.62 (1.39–1.90)***	1.39 (1.20–1.63)***	1.31 (1.12–1.53)***	1.35 (1.16—1.58)***
Never married	1.52 (1.47–1.58)***	1.50 (1.44–1.55)***	1.46 (1.41–1.51)***	1.45 (1.39–1.50)***		1.59 (1.51–1.67)***	1.40 (1.34–1.48)***	1.34 (1.27–1.40)***	1.35 (1.28–1.42)***
Median income									
< $35,000	1.00 (reference)	1.00 (reference)	1.00 (reference)	1.00 (reference)		1.00 (reference)	1.00 (reference)	1.00 (reference)	1.00 (reference)
$35,000–$44,999	0.87 (0.80–0.96)**	0.85 (0.77–0.93)*	0.89 (0.81–0.98)*	0.89 (0.81–0.98)*		0.83 (0.72–0.95)**	0.86 (0.75–0.99)^*^	0.93 (0.81–1.07)^ns^	0.92 (0.80–1.06)^ns^
$45,000–$54,999	0.79 (0.72–0.86)***	0.83 (0.76–0.91)**	0.87 (0.79–0.95)**	0.87 (0.79—0.95)**		0.74 (0.65–0.84)***	0.87 (0.76–1.00)^ns^	0.95 (0.83–1.09)^ns^	0.94 (0.82–1.09)^ns^
$55,000–$64,999	0.67 (0.61–0.73)***	0.78 (0.71–0.85)***	0.79 (0.72–0.87)***	0.79 (0.72–0.87)***		0.68 (0.60–0.77)***	0.83 (0.73–0.96)*	0.93 (0.81—1.07)^ns^	0.92 (0.80–1.06)^ns^
$65,000–$74,999	0.61 (0.56–0.66)***	0.73 (0.66–0.80)***	0.76 (0.69–0.83)***	0.76 (0.69–0.83)***		0.57 (0.50–0.65)***	0.75 (0.65–0.87)***	0.83 (0.71–0.96)*	0.83 (0.71–0.96)*
> $75,000	0.53 (0.48–0.57)***	0.67 (0.61–0.74)***	0.70 (0.63–0.77)***	0.70 (0.63–0.77)***		0.50 (0.44–0.57)***	0.69 (0.60–0.81)***	0.78 (0.67–0.90)***	0.77 (0.66–0.89)***
Urban–Rural									
Counties in metropolitan areas greater than 1 million	1.00 (reference)	1.00 (reference)	1.00 (reference)	1.00 (reference)		1.00 (reference)	1.00 (reference)	1.00 (reference)	1.00 (reference)
Counties in metropolitan areas of 250 k to 1 million	1.07 (1.03–1.10)***	1.02 (1.01–1.07)^ns^	1.02 (0.99–1.06)*	1.04 (1.01–1.07)*		0.97 (0.93–1.02)^ns^	0.97 (0.91–1.01)^ns^	0.96 (0.91–1.00)^ns^	0.97 (0.92–1.02)^ns^
Counties in metropolitan areas less than 250 k	1.26 (1.21–1.32)***	1.06 (1.01–1.11)*	1.08 (1.03–1.13)**	1.08 (1.03–1.14)**		1.16 (1.08–1.24)***	1.01 (0.94–1.09)^ns^	1.03 (0.96–1.11)^ns^	1.03 (0.96–1.12)^ns^
Non-metropolitan counties adjacent to a metropolitan area	1.28 (1.22–1.34)***	1.03 (0.97–1.08)^ns^	1.04 (0.99–1.10)^ns^	1.05 (0.99–1.10)^ns^		1.15 (1.06–1.23)***	0.98 (0.90–1.07)^ns^	0.99 (0.91–1.08)^ns^	1.00 (0.92–1.09)^ns^
Non-metropolitan counties non-adjacent to a metropolitan area	1.37 (1.30–1.45)***	1.08 (1.02–1.15)*	1.09 (1.03–1.16)*	1.07 (1.00–1.14)*		1.30 (1.20–1.41)***	1.11 (1.01–1.22)	1.13 (1.03–1.25)*	1.12 (1.02–1.23)*
Radiation therapy									
No	1.00 (reference)	–	–	1.00 (reference)		1.00 (reference)	–	–	1.00 (reference)
Yes	0.53 (0.52–0.55)***	–	–	0.69 (0.67–0.71)***		0.66 (0.63–0.68)***	–	–	0.81 (0.77–0.84)***
Chemotherapy									
No	1.00 (reference)	–	–	1.00 (reference)		1.00 (reference)	–	–	1.00 (reference)
Yes	0.95 (0.92–0.97)***	–	–	0.93 (0.90–0.96)***		2.44 (2.36–2.54)***	–	–	1.16 (1.11–1.22)***

For the *p*-value, ns indicates not significant, *p < 0.05, **p < 0.01, ***p < 0.001

*HER2* Human epidermal growth factor receptor 2, *Model 1* adjusting for sociodemographic factors, *Model 2* adjusted for sociodemographic and tumor factors; and *Model 3* adjusted for radiation therapy and chemotherapy in addition to those variables in the second model

#### Breast cancer-specific mortality

Women who underwent BCS had a significantly lower hazard of breast cancer-specific mortality as compared to women who refused the recommended treatment [HR 0.14 (95% CI 0.13–0.16)], after adjusting for covariates (model 3). Women who underwent mastectomy were also significantly less likely to die of breast cancer than women who refused the recommended surgery (HR 0.21, 95% CI 0.19–0.23) in model 3. Similar to that of all-cause mortality, non-Hispanic Black women had the highest crude hazard of breast cancer mortality (HR 1.88, 95% CI 1.80–1.98). After adjusting for surgery type and other covariates (model 3), the hazard ratio of breast cancer-specific mortality remained higher among non-Hispanic Blacks, although not statistically significant (HR 1.13, 95% CI 0.90–1.42). In model 3, women with HER2-enriched and triple-negative tumors were significantly more likely to die of breast cancer as compared to luminal A with hazard ratios of 5.31 (95% CI 3.82–7.40) and 12.93 (95% CI 10.62–15.73), respectively. A significantly higher hazard ratios of breast cancer-specific mortality were observed in those with grade III cancer, regional tumors with both direct and lymph node involvement, tumors located in the lower-inner quadrant of the breast, and 80 years or more.

### Sensitivity analysis

Results from the six sensitivity analyses were consistent in magnitude and direction with the findings in Tables 3 and 4. In the first 3 sensitivity analyses (Supplemental Tables S1–S3) that excluded patients 40–49 years old, patients 40–59, and patients with pathological inflammatory breast cancer, race was significantly associated with refusal of recommended surgery. In the last 3 sensitivity analyses (Supplemental Tables S4–S6) that excluded patients 40–49 years old, patients 40–59, and patients with pathological inflammatory breast cancer, surgery type was significantly associated with breast cancer-related mortality.

## Discussion

Racial disparities in access to healthcare, including breast cancer treatment, have been widely reported in the USA [26–28]. In this nation-wide large cohort of women aged 40 years and above with a primary diagnosis of breast cancer in the USA from 2010 to 2017, we sought to assess racial and ethnic disparities in undergoing the recommended surgery. The findings from our study suggest that race/ethnicity, increasing age, tumor subtypes, income, area of residence (rural vs. urban), and year of diagnosis are significant predictors of whether the recommended surgery for breast cancer was performed for women aged 40 years and older.

Non-Hispanic Blacks were more likely to refuse getting surgery as recommended for breast cancer compared to Non-Hispanic Whites. This is consistent with similar studies that have assessed the predictors of breast cancer-recommended surgery refusal [7, 14–19]. This finding can be attributed to low socioeconomic status (SES) and distrust of health care among racial minorities [7, 27, 29]. Evidence suggests that income and employment are major determinants of a person’s refusal to undergo recommended surgery [7, 30]. This is partly explained in that people with low income may not have health insurance or the financial resources to cover the cost of treatment or any additional out-of-pocket expenses required for a recommended surgery for breast cancer [7, 30]. However, contrary to what has been previously documented in other studies, higher median household income (> 75,000) at the census tract level was significantly associated with an increased odds of refusing the recommended surgery in our study. The reason for this finding is unclear. Additionally, this study is limited since we did not have access to insurance coverage and employment status data for different racial and ethnic groups. This study also revealed that non-Hispanic Black women were diagnosed at more advanced stages of breast cancer for which surgery may not be curative. This is similar to a study that found that NH Black women were 3 times (OR 3.00 [95% CI, 2.41 to 3.75]) more likely to not undergo mammography screening and 2.5 times (OR 2.49 [CI 1.59 to 3.92]) more likely to present with a more advanced stage of breast cancer at diagnosis as compared to White women [28]. For persons who present with more advanced stages of breast cancer for which surgery may not be curative, a decision to undergo a recommended surgery may not seem favorable especially when it weighed against the economic burden following the procedure.

Increasing age was also significantly associated with an increased odds of refusing recommended surgery being performed, which was disproportionate for women aged 80 years and older compared to those aged 40–49 years consistent with other studies on the topic [7, 14–19]. Advanced age is associated with an increased risk for comorbidities and decreased survival after cancer treatment [4, 31]. As such, these patients may not have had the recommended surgery performed because they may be sicker or have a decreased life expectancy from other comorbidities or from advanced age. Additionally, breast cancer screening after the age of 74 years is controversial and not recommended especially for average-risk older women with less than 10-year life expectancy [31–33]. Hence, women with breast cancer after 70 years could present with an advanced stage of breast cancer or may refuse surgery as this treatment may not impact life expectancy. We were unable to assess the relationship between advanced age, comorbidities, and likelihood of undergoing recommended surgery because the publicly available SEER data do not include clinical information on comorbid conditions.

This study also explored the extent to which disparities in receipt of breast cancer surgery were due to molecular subtype characteristics in an ethnically diverse cohort of older women in the USA. Our study revealed that women with breast cancer subtypes luminal B and HER2 enriched and triple negative as compared to luminal A subtype were more likely to refuse the recommended breast cancer surgery. This was particularly true for HER2-enriched tumors and triple-negative tumors, which have been more closely associated with aggressive tumor features, such as increased tumor size, positive lymph nodes, and lymphovascular invasion [34]. Although different negative prognostic factors exist for different breast cancer subtypes, studies have found that cases with HER2 and triple-negative tumors, predominantly diagnosed among racial minorities, such as African American and American Indian/ Native Alaska women, have the worst 5-year survival and overall survival among the other subtypes [34, 35]. As such, cases with more aggressive subtypes such as HER2 enriched may likely present at more advanced stages of breast cancer at diagnosis for which surgery may not be curative and this may impact the decision to pursue a recommended surgery for breast cancer.

Similar to other studies, women who received a recommended surgery for breast cancer treatment had a better survival probability as compared to those who refused the recommended treatment [4, 8]. Significant predictors of death following a breast cancer diagnosis included age 80 years or older, HER2-enriched and triple-negative tumors, and tumor features suggestive of advanced disease, such as tumor grade III and lymph node involvement. As previously discussed, HER2-enriched and triple-negative tumors, which were mostly present among African American women, were associated with aggressive disease [34–37]. This suggests that breast tumor subtypes’ impact on breast cancer disease progression may be a significant contributor of whether a recommended surgery is refused or performed and could ultimately affect survival benefit from breast cancer surgery particularly among NH Black women.

The study strengths include the use of the SEER database, a large nationally representative cancer registry which allows for generalizability of results to the general population in the USA. Additionally, to our knowledge, this is one of the few studies to examine the refusal of surgery for breast cancer specifically by examining and adjusting for tumor stage, grade, and molecular subtype.

There are several limitations in this study. First, the information on chemotherapy and radiation therapy were incomplete, thus their interpretations should be done with caution. Second, it is possible that an eligible patient with recommended surgery purposely chose to have a surgery outside SEER areas or abroad. This has a potential of introducing differential bias in the analysis. Third, the SEER database lacks the detailed clinical and circumstantial information for each patient. There is therefore the potential for residual confounding as we were limited in our ability to control for other potential confounders, such as comorbidities. Furthermore, the lack of detailed clinical information limits our ability to fully understand the reasons why a recommended surgery for breast cancer treatment was not performed. Fourth, there is the potential for selection bias since individuals who were excluded from the analysis could be significantly different in examined characteristics from those included in the analysis. Fifth, inadequate health insurance status is documented to be an important underlying reason for refusal to undergo surgical treatment [7, 13], but we did not have information on health insurance coverage and employment status in this SEER dataset, and hence, we cannot assess the effect of health insurance status on the refusal of surgery for different racial and ethnic groups. Lastly, SEER does not have variables assessing social factors, such as inadequate care for children/grandchildren, lack of requisite transportation, or peer support. These factors may impact one’s decision to refuse or accept the recommended surgery.

## Conclusion

In conclusion, this study highlights the racial and ethnic disparities in the receipt of a recommended surgery for breast cancer treatment in the United States. Although NH Black women are disproportionately affected by breast cancer, they are more likely to refuse a recommended surgery for breast cancer treatment even when it is the main modality of treatment. Refusal of surgery for breast cancer treatment can negatively impact survival in this vulnerable group. Physicians should be cognizant of the predictors of surgery refusal. Also, targeted and culturally relevant interventions should be implemented for this at-risk population.

## Supplementary Information

Below is the link to the electronic supplementary material.

Supplementary file1 (DOCX 60 kb)

## Data Availability

The datasets generated during and/or analyzed during the current study are not publicly available due to SEER’s Data use agreement and terms. The data access requests should be directly made to SEER https://service.cancer.gov/seer-data-access
