# Supplementary material for: Racial and ethnic disparities in the refusal of surgical treatment in women 40 years and older with breast cancer in the USA between 2010 and 2017
Source: Breast Cancer Res Treat. 2022 Jun 24;194(3):643–61. doi: 10.1007/s10549-022-06653-w (PMC9287205; doi:10.1007/s10549-022-06653-w)
Supplement: Supplementary file 1 — Supplementary file1 (DOCX 60 kb) [file 10549_2022_6653_MOESM1_ESM.docx]

**Supplemental Tables**

**Table S1. Multivariable logistic regression analyses of factors associated with refusing to undergo surgery as recommend among US. Women 50 or above with breast cancer diagnosis between 2010-2017**

| **Variable** | **Adjusted Odds Ratio  of refusing the recommended surgery** | **[95% Confidence Interval]** | | **Sig** |
| --- | --- | --- | --- | --- |
| Race and ethnicity | | | | |
| Non-Hispanic White | 1.00 (reference) | . | . |  |
| Non-Hispanic Black | 2.104 | 1.793 | 2.469 | *** |
| Hispanic (All races) | .887 | .721 | 1.09 |  |
| Other | 1.053 | .862 | 1.287 |  |
| Subtypes | | | | |
| Luminal A | 1.00 (reference) | . | . |  |
| Luminal B | 1.823 | 1.518 | 2.19 | *** |
| HER2 enriched | 1.62 | 1.211 | 2.167 | *** |
| Triple negative | 1.293 | 1.046 | 1.597 | ** |
| Age at diagnosis | | | | |
| 50-59 | 1.00 (reference) | . | . |  |
| 60-69 | .9 | .75 | 1.081 |  |
| 70-79 | .837 | .69 | 1.015 | * |
| 80+ | 2.674 | 2.237 | 3.197 | *** |
| Tumor grade | | | | |
| Grade I; well differentiated | 1.00 (reference) | . | . |  |
| Grade II; moderately differentiated | 1.279 | 1.109 | 1.475 | *** |
| Grade III; poorly differentiated | 1.075 | .897 | 1.288 |  |
| Tumor site | | | | |
| Nipple | 1.00 (reference) | . | . |  |
| Central portion of the breast | 1.72 | .822 | 3.6 |  |
| Upper-inner quadrant of the breast | 1.748 | .839 | 3.639 |  |
| Lower-inner quadrant of the breast | 1.483 | .698 | 3.149 |  |
| Upper-outer quadrant of the breast | 1.707 | .828 | 3.521 |  |
| Lower-outer quadrant of the breast | 1.512 | .718 | 3.185 |  |
| Axillary tail of the breast | 1.611 | .539 | 4.816 |  |
| Overlapping lesion of the breast | 1.827 | .885 | 3.77 |  |
| Tumor stage | | | | |
| Localized only | 1.00 (reference) | . | . |  |
| Regional, direct extension only | 4.321 | 3.478 | 5.368 | *** |
| Regional, lymph nodes only | 1.531 | 1.323 | 1.773 | *** |
| Regional, both direct extension and lymph nodes | 3.937 | 3.147 | 4.926 | *** |
| Chemotherapy | | | | |
| No | 1.00 (reference) | . | . |  |
| Yes | .196 | .157 | .245 | *** |
| Radiation | | | | |
| No | 1.00 (reference) | . | . |  |
| Yes | .015 | .01 | .023 | *** |
| Year of diagnosis | | | | |
| 2010 | 1.00 (reference) | . | . |  |
| 2011 | 1.218 | .924 | 1.606 |  |
| 2012 | 1.482 | 1.138 | 1.929 | *** |
| 2013 | 1.477 | 1.135 | 1.921 | *** |
| 2014 | 1.635 | 1.261 | 2.12 | *** |
| 2015 | 2.113 | 1.651 | 2.705 | *** |
| 2016 | 2.309 | 1.807 | 2.95 | *** |
| 2017 | 2.309 | 1.809 | 2.949 | *** |
| Median income | | | | |
| < $35,000 | 1.00 (reference) | . | . |  |
| $35,000-44,999 | 1.067 | .612 | 1.858 |  |
| $45,000-54,999 | 1.485 | .863 | 2.555 |  |
| $55,000-64,999 | 1.214 | .699 | 2.107 |  |
| $65,000-74,999 | 1.495 | .856 | 2.613 |  |
| >$75,000 | 1.879 | 1.079 | 3.273 | ** |
| Urban-Rural | | | | |
| Counties in metropolitan areas greater than 1 million | 1.00 (reference) | . | . |  |
| Counties in metropolitan areas of 250k to 1 million | 1.179 | 1.023 | 1.358 | ** |
| Counties in metropolitan areas less than 250k | .935 | .728 | 1.202 |  |
| Nonmetropolitan counties adjacent to a metropolitan area | .988 | .744 | 1.313 |  |
| Nonmetropolitan counties non-adjacent to a metropolitan area | 1.046 | .758 | 1.442 |  |
| Marital status | | | | |
| Married | 1.00 (reference) | . | . |  |
| Unmarried/Domestic partner | .5 | .069 | 3.601 |  |
| Divorced | 1.769 | 1.472 | 2.126 | *** |
| Widowed | 1.668 | 1.435 | 1.938 | *** |
| Separated | 2.127 | 1.304 | 3.468 | *** |
| Never married | 1.901 | 1.604 | 2.253 | *** |
| *Sig: Statistically significant*  **** p<.01, ** p<.05, * p<.1* | | | | |

**Table S2. Multivariable logistic regression analyses of factors associated with refusing to undergo surgery as recommend among US. Women 60 or above with breast cancer diagnosis between 2010-2017**

| **Variables** | **Adjusted Odds Ratio  of refusing the recommended surgery** | **[95% Confidence Interval]** | | **Sig** |
| --- | --- | --- | --- | --- |
| Race and ethnicity | | | | |
| Non-Hispanic White | 1.00 (reference) | . | . |  |
| Non-Hispanic Black | 2.036 | 1.703 | 2.435 | *** |
| Hispanic (All races) | .894 | .707 | 1.132 |  |
| Other | .893 | .701 | 1.138 |  |
| Subtypes | | | | |
| Luminal A | 1.00 (reference) | . | . |  |
| Luminal B | 1.675 | 1.355 | 2.071 | *** |
| HER2 enriched | 1.233 | .852 | 1.784 |  |
| Triple negative | 1.217 | .959 | 1.544 |  |
| Age at diagnosis | | | | |
| 60-69 | 1.00 (reference) | . | . |  |
| 70-79 | .934 | .775 | 1.126 |  |
| 80+ | 3.06 | 2.581 | 3.628 | *** |
| Tumor grade | | | | |
| Grade I; well differentiated | 1.00 (reference) | . | . |  |
| Grade II; moderately differentiated | 1.227 | 1.054 | 1.429 | *** |
| Grade III; poorly differentiated | .956 | .783 | 1.168 |  |
| Tumor site | | | | |
| Nipple | 1.00 (reference) | . | . |  |
| Central portion of the breast | 2.358 | .939 | 5.921 | * |
| Upper-inner quadrant of the breast | 2.428 | .971 | 6.071 | * |
| Lower-inner quadrant of the breast | 2.182 | .857 | 5.554 |  |
| Upper-outer quadrant of the breast | 2.501 | 1.01 | 6.196 | ** |
| Lower-outer quadrant of the breast | 1.985 | .783 | 5.033 |  |
| Axillary tail of the breast | 2.236 | .623 | 8.03 |  |
| Overlapping lesion of the breast | 2.577 | 1.04 | 6.388 | ** |
| Tumor stage | | | | |
| Localized only | 1.00 (reference) | . | . |  |
| Regional, direct extension only | 4.169 | 3.314 | 5.244 | *** |
| Regional, lymph nodes only | 1.42 | 1.199 | 1.68 | *** |
| Regional, both direct extension and lymph nodes | 3.693 | 2.884 | 4.729 | *** |
| Chemotherapy | | | | |
| No | 1.00 (reference) | . | . |  |
| Yes | .242 | .183 | .32 | *** |
| Radiation | | | | |
| No | 1.00 (reference) | . | . |  |
| Yes | .016 | .01 | .026 | *** |
| Year of diagnosis | | | | |
| 2010 | 1.00 (reference) | . | . |  |
| 2011 | 1.177 | .862 | 1.606 |  |
| 2012 | 1.605 | 1.2 | 2.147 | *** |
| 2013 | 1.626 | 1.217 | 2.171 | *** |
| 2014 | 1.573 | 1.174 | 2.108 | *** |
| 2015 | 2.236 | 1.7 | 2.94 | *** |
| 2016 | 2.259 | 1.718 | 2.971 | *** |
| 2017 | 2.407 | 1.834 | 3.158 | *** |
| Median income | | | | |
| < $35,000 | 1.00 (reference) | . | . |  |
| $35,000-44,999 | 1.067 | .59 | 1.929 |  |
| $45,000-54,999 | 1.366 | .764 | 2.445 |  |
| $55,000-64,999 | 1.016 | .561 | 1.839 |  |
| $65,000-74,999 | 1.287 | .707 | 2.343 |  |
| $75,000+ | 1.649 | .909 | 2.991 | * |
| Urban-Rural | | | | |
| Counties in metropolitan areas greater than 1 million | 1.00 (reference) | . | . |  |
| Counties in metropolitan areas of 250k to 1 million | 1.13 | .967 | 1.32 |  |
| Counties in metropolitan areas less than 250k | .776 | .583 | 1.033 | * |
| Nonmetropolitan counties adjacent to a metropolitan area | .969 | .719 | 1.305 |  |
| Nonmetropolitan counties non-adjacent to a metropolitan area | .855 | .598 | 1.223 |  |
| Marital status | | | | |
| Married | 1.00 (reference) | . | . |  |
| Unmarried/Domestic partner | .748 | .102 | 5.48 |  |
| Divorced | 1.862 | 1.513 | 2.292 | *** |
| Widowed | 1.665 | 1.42 | 1.952 | *** |
| Separated | 1.826 | .949 | 3.511 | * |
| Never married | 1.875 | 1.531 | 2.296 | *** |
| *Sig: Statistically significant*  **** p<.01, ** p<.05, * p<.1* | | | | |

**Table S3. Multivariable logistic regression analyses of factors associated with refusing to undergo surgery as recommend among US. Women 40 or above with non-pathological inflammatory breast cancer diagnosis between 2010-2017**

| **Variable** | **Adjusted Odds Ratio  of refusing the recommended surgery** | **[95% Confidence Interval]** | | **Sig** |
| --- | --- | --- | --- | --- |
| Race and ethnicity | | | | |
| Non-Hispanic White | 1.00 (reference) | . | . |  |
| Non-Hispanic Black | 2.123 | 1.823 | 2.473 | *** |
| Hispanic (All races) | .973 | .808 | 1.171 |  |
| Other | 1.181 | .986 | 1.416 | * |
| Subtypes | | | | |
| Luminal A | 1.00 (reference) | . | . |  |
| Luminal B | 1.925 | 1.622 | 2.284 | *** |
| HER2 enriched | 1.642 | 1.246 | 2.164 | *** |
| Triple negative | 1.362 | 1.115 | 1.662 | *** |
| Age at diagnosis | | | | |
| 40-49 | 1.00 (reference) | . | . |  |
| 50-59 | 1.152 | .93 | 1.427 |  |
| 60-69 | 1.04 | .839 | 1.288 |  |
| 70-79 | .965 | .772 | 1.206 |  |
| 80+ | 3.062 | 2.478 | 3.783 | *** |
| Tumor grade | | | | |
| Grade I; well differentiated | 1.00 (reference) | . | . |  |
| Grade II; moderately differentiated | 1.305 | 1.138 | 1.497 | *** |
| Grade III; poorly differentiated | 1.114 | .937 | 1.324 |  |
|  | | | | |
| Nipple | 1.00 (reference) | . | . |  |
| Central portion of the breast | 1.726 | .827 | 3.603 |  |
| Upper-inner quadrant of the breast | 1.851 | .893 | 3.839 | * |
| Lower-inner quadrant of the breast | 1.54 | .729 | 3.254 |  |
| Upper-outer quadrant of the breast | 1.778 | .865 | 3.657 |  |
| Lower-outer quadrant of the breast | 1.567 | .748 | 3.285 |  |
| Axillary tail of the breast | 1.751 | .615 | 4.99 |  |
| Overlapping lesion of the breast | 1.923 | .935 | 3.957 | * |
| Tumor stage | | | | |
| Localized only | 1.00 (reference) | . | . |  |
| Regional, direct extension only | 4.203 | 3.389 | 5.212 | *** |
| Regional, lymph nodes only | 1.574 | 1.372 | 1.806 | *** |
| Regional, both direct extension and lymph nodes | 4.015 | 3.234 | 4.984 | *** |
| Chemotherapy | | | | |
| No | 1.00 (reference) | . | . |  |
| Yes | .166 | .135 | .204 | *** |
| Radiation | | | | |
| No | 1.00 (reference) | . | . |  |
| Yes | .015 | .01 | .022 | *** |
| Year of diagnosis | | | | |
| 2010 | 1.00 (reference) | . | . |  |
| 2011 | 1.262 | .973 | 1.637 | * |
| 2012 | 1.508 | 1.176 | 1.935 | *** |
| 2013 | 1.454 | 1.133 | 1.867 | *** |
| 2014 | 1.607 | 1.255 | 2.058 | *** |
| 2015 | 2.094 | 1.657 | 2.646 | *** |
| 2016 | 2.256 | 1.788 | 2.848 | *** |
| 2017 | 2.253 | 1.786 | 2.842 | *** |
| Median income | | | | |
| < $35,000 | 1.00 (reference) | . | . |  |
| $35,000-44,999 | 1.083 | .632 | 1.855 |  |
| $45,000-54,999 | 1.516 | .895 | 2.566 |  |
| $55,000-64,999 | 1.204 | .705 | 2.055 |  |
| $65,000-74,999 | 1.478 | .861 | 2.539 |  |
| $75,000+ | 1.847 | 1.079 | 3.162 | ** |
| Urban-Rural | | | | |
| Counties in metropolitan areas greater than 1 million | 1.00 (reference) | . | . |  |
| Counties in metropolitan areas of 250k to 1 million | 1.168 | 1.02 | 1.337 | ** |
| Counties in metropolitan areas less than 250k | .908 | .712 | 1.159 |  |
| Nonmetropolitan counties adjacent to a metropolitan area | .959 | .728 | 1.265 |  |
| Nonmetropolitan counties non-adjacent to a metropolitan area | 1.024 | .75 | 1.399 |  |
| Marital status | | | | |
| Married | 1.00 (reference) | . | . |  |
| Unmarried/Domestic partner | .395 | .055 | 2.837 |  |
| Divorced | 1.689 | 1.417 | 2.015 | *** |
| Widowed | 1.661 | 1.434 | 1.925 | *** |
| Separated | 2.789 | 1.876 | 4.147 | *** |
| Never married | 1.882 | 1.61 | 2.199 | *** |
| *Sig: Statistically significant*  **** p<.01, ** p<.05, * p<.1* | | | | |

**Table S4. Multivariable Cox regression analyses of factors associated with all-cause mortality and breast cancer-related mortality among US. Women 50 or above with breast cancer between 2010-2017**

| **Variable** | **Adjusted proportional Hazard Ratio** | **[95% Confidence Interval]** | | **Sig** |
| --- | --- | --- | --- | --- |
| Surgery type | | | | |
| No surgery | 1.00 (reference) | . | . |  |
| Breast conservatory surgery (BCS) | .15 | .133 | .169 | *** |
| Mastectomy | .215 | .192 | .241 | *** |
| Race and ethnicity | | | | |
| Non-Hispanic White | 1.00 (reference) | . | . |  |
| Non-Hispanic Black | 1.113 | .871 | 1.423 |  |
| Hispanic (All races) | .804 | .587 | 1.101 |  |
| Other | .478 | .321 | .711 | *** |
| Subtypes | | | | |
| Luminal A | 1.00 (reference) | . | . |  |
| Luminal B | 1.742 | 1.253 | 2.42 | *** |
| HER2 enriched | 4.824 | 3.404 | 6.836 | *** |
| Triple negative | 11.358 | 9.22 | 13.992 | *** |
| Age at diagnosis | | | | |
| 50-59 | 1.00 (reference) | . | . |  |
| 60-69 | 1.744 | 1.348 | 2.255 | *** |
| 70-79 | 4.524 | 3.476 | 5.888 | *** |
| 80+ | 9.913 | 7.592 | 12.942 | *** |
| Tumor grade | | | | |
| Grade I; well differentiated | 1.00 (reference) | . | . |  |
| Grade II; moderately differentiated | 2.015 | 1.852 | 2.191 | *** |
| Grade III; poorly differentiated | 4.149 | 3.807 | 4.522 | *** |
| Tumor site | | | | |
| Nipple | 1.00 (reference) | . | . |  |
| Central portion of the breast | 1.161 | .886 | 1.521 |  |
| Upper-inner quadrant of the breast | 1.242 | .951 | 1.624 |  |
| Lower-inner quadrant of the breast | 1.32 | 1.005 | 1.733 | ** |
| Upper-outer quadrant of the breast | 1.136 | .873 | 1.478 |  |
| Lower-outer quadrant of the breast | 1.238 | .945 | 1.621 |  |
| Axillary tail of the breast | 1.208 | .833 | 1.752 |  |
| Overlapping lesion of the breast | 1.176 | .903 | 1.531 |  |
| Tumor stage | | | | |
| Localized only | 1.00 (reference) | . | . |  |
| Regional, direct extension only | 3.39 | 3.03 | 3.792 | *** |
| Regional, lymph nodes only | 3.201 | 3.049 | 3.36 | *** |
| Regional, both direct extension and lymph nodes | 6.87 | 6.404 | 7.37 | *** |
| Chemotherapy | | | | |
| No | 1.00 (reference) | . | . |  |
| Yes | 1.168 | 1.108 | 1.231 | *** |
| Radiation | | | | |
| No | 1.00 (reference) | . | . |  |
| Yes | .769 | .734 | .806 | *** |
| Median income | | | | |
| < $35,000 | 1.00 (reference) | . | . |  |
| $35,000-44,999 | .934 | .799 | 1.091 |  |
| $45,000-54,999 | .957 | .82 | 1.118 |  |
| $55,000-64,999 | .924 | .789 | 1.083 |  |
| $65,000-74,999 | .84 | .714 | .988 | ** |
| $75,000+ | .778 | .661 | .914 | *** |
| Urban-Rural | | | | |
| Counties in metropolitan areas greater than 1 million | 1.00 (reference) | . | . |  |
| Counties in metropolitan areas of 250k to 1 million | .956 | .906 | 1.009 |  |
| Counties in metropolitan areas less than 250k | 1.019 | .938 | 1.107 |  |
| Nonmetropolitan counties adjacent to a metropolitan area | .972 | .885 | 1.067 |  |
| Nonmetropolitan counties non-adjacent to a metropolitan area | 1.108 | .998 | 1.23 | * |
|  | | | | |
| Married | 1.00 (reference) | . | . |  |
| Unmarried/Domestic partner | 1.516 | 1.023 | 2.246 | ** |
| Divorced | 1.266 | 1.188 | 1.348 | *** |
| Widowed | 1.296 | 1.223 | 1.372 | *** |
| Separated | 1.322 | 1.097 | 1.592 | *** |
| Never Married | 1.36 | 1.281 | 1.444 | *** |
| *Sig: Statistically significant*  **** p<.01, ** p<.05, * p<.1* | | | | |

**Table S5. Multivariable Cox regression analyses of factors associated with all-cause mortality and breast cancer-related mortality among US. Women 60 or above with breast cancer between 2010-2017**

**Regression results**

| **Variable** | **Adjusted proportional Hazard Ratio** | **[95% Confidence Interval]** | | **Sig** |
| --- | --- | --- | --- | --- |
| Surgery type | | | | |
| No surgery | 1.00 (reference) | . | . |  |
| Breast conservatory surgery (BCS) | .163 | .143 | .187 | *** |
| Mastectomy | .231 | .204 | .262 | *** |
| Race and ethnicity | | | | |
| Non-Hispanic White | 1.00 (reference) | . | . |  |
| Non-Hispanic Black | .995 | .741 | 1.336 |  |
| Hispanic (All races) | .788 | .541 | 1.148 |  |
| Other | .591 | .376 | .93 | ** |
| Subtypes | | | | |
| Luminal A | 1.00 (reference) | . | . |  |
| Luminal B | 1.548 | 1.068 | 2.242 | ** |
| HER2 enriched | 5.376 | 3.674 | 7.864 | *** |
| Triple negative | 8.433 | 6.659 | 10.679 | *** |
| Age at diagnosis | | | | |
| : base 60-69 | 1.00 (reference) | . | . |  |
| 70-79 | 2.451 | 1.904 | 3.156 | *** |
| 80+ | 5.149 | 3.986 | 6.652 | *** |
| Tumor grade | | | | |
| Grade I; well differentiated | 1.00 (reference) | . | . |  |
| Grade II; moderately differentiated | 1.903 | 1.733 | 2.088 | *** |
| Grade III; poorly differentiated | 3.823 | 3.471 | 4.212 | *** |
| Tumor site | | | | |
| Nipple | 1.00 (reference) | . | . |  |
| Central portion of the breast | 1.191 | .872 | 1.626 |  |
| Upper-inner quadrant of the breast | 1.354 | .995 | 1.844 | * |
| Lower-inner quadrant of the breast | 1.37 | 1 | 1.876 | * |
| Upper-outer quadrant of the breast | 1.217 | .898 | 1.648 |  |
| Lower-outer quadrant of the breast | 1.364 | .999 | 1.862 | * |
| Axillary tail of the breast | 1.162 | .737 | 1.831 |  |
| Overlapping lesion of the breast | 1.247 | .92 | 1.69 |  |
| Tumor stage | | | | |
| Localized only | 1.00 (reference) | . | . |  |
| Regional, direct extension only | 3.35 | 2.957 | 3.796 | *** |
| Regional, lymph nodes only | 3.101 | 2.926 | 3.286 | *** |
| Regional, both direct extension and lymph nodes | 6.528 | 6.002 | 7.1 | *** |
| Chemotherapy | | | | |
| No | 1.00 (reference) | . | . |  |
| Yes | 1.118 | 1.051 | 1.189 | *** |
| Radiation | | | | |
| No | 1.00 (reference) | . | . |  |
| Yes | .703 | .664 | .744 | *** |
| Median income | | | | |
| < $35,000 | 1.00 (reference) | . | . |  |
| $35,000-44,999 | .961 | .795 | 1.161 |  |
| $45,000-54,999 | 1.005 | .833 | 1.213 |  |
| $55,000-64,999 | .952 | .786 | 1.154 |  |
| $65,000-74,999 | .852 | .699 | 1.038 |  |
| $75,000+ | .784 | .644 | .954 | ** |
| Urban-Rural | | | | |
| Counties in metropolitan areas greater than 1 million | 1.00 (reference) | . | . |  |
| Counties in metropolitan areas of 250k to 1 million | .956 | .896 | 1.02 |  |
| Counties in metropolitan areas less than 250k | 1.007 | .913 | 1.112 |  |
| Nonmetropolitan counties adjacent to a metropolitan area | .963 | .863 | 1.075 |  |
| Nonmetropolitan counties non-adjacent to a metropolitan area | 1.091 | .966 | 1.234 |  |
| Marital Status | | | | |
| Married | 1.00 (reference) | . | . |  |
| Unmarried/Domestic partner | 1.322 | .731 | 2.39 |  |
| Divorced | 1.331 | 1.232 | 1.437 | *** |
| Widowed | 1.29 | 1.213 | 1.373 | *** |
| Separated | 1.458 | 1.123 | 1.892 | *** |
| Never Married | 1.312 | 1.212 | 1.42 | *** |
| *Sig: Statistically significant*  **** p<.01, ** p<.05, * p<.1* | | | | |

**Table S6. Multivariable Cox regression analyses of factors associated with all-cause mortality and breast cancer-related mortality among US. Women 40 or above with non-pathological inflammatory breast cancer between 2010-2017**

| **Variable** | **Adjusted proportional Hazard Ratio** | **[95% Confidence Interval]** | | **Sig** |
| --- | --- | --- | --- | --- |
| Surgery type | | | | |
| No surgery | 1.00 (reference) | . | . |  |
| Breast conservatory surgery (BCS) | .144 | .128 | .161 | *** |
| Mastectomy | .206 | .185 | .229 | *** |
| Race and ethnicity | | | | |
| Non-Hispanic White | 1.00 (reference) | . | . |  |
| Non-Hispanic Black | 1.131 | .9 | 1.42 |  |
| Hispanic (All races) | .828 | .624 | 1.099 |  |
| Other | .473 | .326 | .686 | *** |
|  | | | | |
| : base Luminal A | 1.00 (reference) | . | . |  |
| Luminal B | 1.598 | 1.164 | 2.194 | *** |
| HER2 enriched | 5.351 | 3.841 | 7.454 | *** |
| Triple negative | 12.959 | 10.642 | 15.78 | *** |
| Age at diagnosis | | | | |
| 40-49 | 1.00 (reference) | . | . |  |
| 50-59 | .919 | .687 | 1.229 |  |
| 60-69 | 1.653 | 1.237 | 2.209 | *** |
| 70-79 | 4.483 | 3.338 | 6.021 | *** |
| 80+ | 10.052 | 7.467 | 13.533 | *** |
| Tumor grade | | | | |
| Grade I; well differentiated | 1.00 (reference) | . | . |  |
| Grade II; moderately differentiated | 2.085 | 1.924 | 2.26 | *** |
| Grade III; poorly differentiated | 4.422 | 4.074 | 4.8 | *** |
| Tumor site | | | | |
| Nipple | 1.00 (reference) | . | . |  |
| Central portion of the breast | 1.184 | .912 | 1.536 |  |
| Upper-inner quadrant of the breast | 1.273 | .984 | 1.648 | * |
| Lower-inner quadrant of the breast | 1.356 | 1.043 | 1.762 | ** |
| Upper-outer quadrant of the breast | 1.151 | .893 | 1.484 |  |
| Lower-outer quadrant of the breast | 1.234 | .952 | 1.6 |  |
| Axillary tail of the breast | 1.156 | .814 | 1.642 |  |
| Overlapping lesion of the breast | 1.201 | .931 | 1.549 |  |
| Tumor stage | | | | |
| Localized only | 1.00 (reference) | . | . |  |
| Regional, direct extension only | 3.331 | 2.99 | 3.711 | *** |
| Regional, lymph nodes only | 3.198 | 3.06 | 3.343 | *** |
| Regional, both direct extension and lymph nodes | 7.073 | 6.631 | 7.544 | *** |
| Chemotherapy | | | | |
| No | 1.00 (reference) | . | . |  |
| Yes | 1.161 | 1.106 | 1.22 | *** |
| Radiation | | | | |
| No | 1.00 (reference) | . | . |  |
| Yes | .805 | .771 | .84 | *** |
| Median income | | | | |
| < $35,000 | 1.00 (reference) | . | . |  |
| $35,000-44,999 | .91 | .789 | 1.049 |  |
| $45,000-54,999 | .939 | .815 | 1.081 |  |
| $55,000-64,999 | .91 | .788 | 1.052 |  |
| $65,000-74,999 | .822 | .709 | .953 | *** |
| $75,000+ | .761 | .656 | .882 | *** |
| Urban-Rural | | | | |
| Counties in metropolitan areas greater than 1 million | 1.00 (reference) | . | . |  |
| Counties in metropolitan areas of 250k to 1 million | .968 | .922 | 1.017 |  |
| Counties in metropolitan areas less than 250k | 1.036 | .96 | 1.118 |  |
| Nonmetropolitan counties adjacent to a metropolitan area | 1 | .917 | 1.09 |  |
| Nonmetropolitan counties non-adjacent to a metropolitan area | 1.121 | 1.018 | 1.236 | ** |
| Marital status | | | | |
| Married | 1.00 (reference) | . | . |  |
| Unmarried/Domestic partner | 1.274 | .89 | 1.824 |  |
| Divorced | 1.238 | 1.169 | 1.312 | *** |
| Widowed | 1.289 | 1.219 | 1.363 | *** |
| Separated | 1.341 | 1.144 | 1.572 | *** |
| Never Married | 1.347 | 1.278 | 1.42 | *** |
| *Sig: Statistically significant*  **** p<.01, ** p<.05, * p<.1* | | | | |
